# Lymphocytic hypophysitis in dogs infected with *Leishmania* spp.

**DOI:** 10.3389/fvets.2023.1208919

**Published:** 2023-09-14

**Authors:** Edenilson Doná Frigerio, Cecilia de Castro Guizelini, Giulia Gonçalves Jussiani, Karen Santos Março, Guilherme Dias de Melo, Tatiane Terumi Negrão Watanabe, Gisele Fabrino Machado

**Affiliations:** ^1^Department of Veterinary Clinics, Surgery and Reproduction, Faculty of Veterinary Medicine, São Paulo State University, UNESP, Araçatuba, Brazil; ^2^Institut Pasteur, Université Paris Cité, Lyssavirus Epidemiology and Neuropathology Unit, Paris, France; ^3^Department Population Health and Pathobiology, College of Veterinary Medicine, North Carolina State University, Raleigh, NC, United States

**Keywords:** pituitary gland, inflammation, T lymphocyte, *Leishmania infantum*, immunohistochemical

## Abstract

**Background:**

Morphological involvement of endocrine glands, such as the pituitary gland, remain uninvestigated in dogs with canine visceral leishmaniasis. Therefore, this study investigated the presence of amastigotes of *Leishmania* spp. and characterized inflammatory changes, highlighting the involvement of TCD3^+^ lymphocytes in different regions of the pituitary gland of dogs.

**Methods:**

Samples were collected from 21 naturally infected dogs and 5 control, uninfected dogs. The different pituitary regions were analyzed in histological sections stained with hematoxylin and eosin (HE) under light microscopy. Inflammation was classified by intensity in a score from 0 to 3, absent (0), mild (1), moderate (2), and marked (3). The immunohistochemical (IHC) evaluation was performed in five high-power fields (hot spot) in a 40x objective of each region with manual counting (Image J1.52ª) of the TCD3^+^ lymphocytes and for amastigotes analyzed in 40x and 100x objectives. The Shapiro–Wilk test was used to assess the normality of the data. Differences between groups were determined by the Mann Whitney test. The correlation between variables was assessed by Sperman’s correlation test. *p* < 0.05 were considered statistically significant.

**Results:**

Amastigotes from the pituitary glands of two infected dogs were identified using IHC. The histopathological evaluation stained with hematoxylin and eosin showed greater intensity of inflammation in the *pars distalis* and *pars intermedia* regions of infected dogs. IHC for TCD3^+^ lymphocytes showed a higher median number of immunolabeled cells in *pars nervosa* in the infected group than in the control group (*p* < 0.05); and expecting a variation in the distribution and number of these cells in naturally infected dogs, the median of the control group was considered a cut-off point, an increase in T lymphocytes (*p* < 0.05) was also observed in the *pars intermedia* and *pars distalis* of an infected subgroup (*n* = 10). A moderate significant correlation between the intensity of inflammation and the number of immunolabeled TCD3^+^ lymphocytes was established in the analyzed pituitary regions, characterizing the occurrence of hypophysitis.

**Conclusion:**

These findings presuppose that inflammation and/or the parasite in the pituitary region can result in gland dysfunction, worsening the clinical condition of the patient and compromising the efficiency of treatment and prognosis.

## 1. Introduction

The hypophysis, or pituitary gland, is a neuroendocrine organ responsible for the interface of communication between the hypothalamus and peripheral endocrine glands, whose regulation is essential for maintaining the homeostasis of organisms in physiological and pathological situations (1, 2). Therefore, systemic pathological changes can lead to dysregulation and dysfunction of the neuroendocrine system (1). The major pituitary disorders are of proliferative origin in dogs (3); however, inflammatory changes have also been reported in humans, with lymphocytic hypophysitis being the most common (4).

Hypophysitis is a chronic inflammatory disorder of primary or secondary origin that occurs in the pituitary gland. Primary hypophysitis is an autoimmune disorder of unknown etiology characterized by inflammation restricted to the pituitary gland, not associated with other systemic inflammatory disorders (4). Secondary hypophysitis is related to local inflammatory processes induced by tumors, cysts, or systemic processes, such as other autoimmune endocrinopathies, medication use, infectious diseases, and other conditions (4, 5).

Several studies have reported primary or secondary hypophysitis promoting endocrine or neurological disorders in dogs; however, many limitations and divergences exist in the studies because it is considered a sporadic condition with the presence of inflammatory infiltrate comprising CD3^+^ T lymphocytes (2, 3, 6–9). Hypophysitis related to bacterial, fungal, viral, or parasitic infectious processes has been poorly studied, although studies in dogs (2, 3), humans (10), and other animals (11) are available. The parasite *Trypanosoma brucei* and *T. congolense* have also been detected by molecular analysis in the brain and pituitary gland of sheep during experimental infection, which is associated with increased plasma cortisol and adrenal gland hyperplasia (11).

The circumventricular organs (CVOs) and choroid plexus can serve as entry routes for parasites and other pathogens to nervous tissue, as demonstrated by infections with the parasite *Trypanosoma brucei* (12). They are devoid of the blood–brain barrier (BBB) but have a blood-liquid barrier formed by occluder junctions between the epithelial cells of the choroid plexus and between the specialized tanycytes lining the ventricular cavities that line the CVOs (13). Neurohypophysis is classified as a CVO and is devoid of effective barriers because it has fenestrated vessels and is susceptible to exposure to circulatory components, such as cytokines, endotoxins, and pathogens, representing an access point that directly influences the central nervous system (CNS). Like other CVOs, neurohypophysis can initiate and modulate local inflammation (12, 14–16).

Leishmaniasis is a zoonotic disease that is distributed worldwide and affects domestic and wild animals (17). The parasite *Leishmania infantum* is the etiological agent of canine visceral leishmaniasis (CanL), listed as one of the most lethal and neglected tropical diseases globally (18, 19). Amastigotes systemically infect the host, causing chronic stimulation of the phagocytic mononuclear system and conferring an immune-mediated character to the pathogenesis of lesions (16), which is directly related to the clinical manifestations (20, 21).

Infected dogs may present with subclinical or systemic diseases, whose clinical signs often include anorexia, hepatosplenomegaly, lymphadenomegaly, and skin lesions (20). In addition, other organs and tissues, such as the thyroid gland (22), heart (23), liver, and kidneys (24), as well as the CNS (25), can be affected. Studies on the pathogenesis of the neurological clinicopathological manifestations caused by *L. infantum* are limited. However, the main neurological pathological findings in these studies were inflammatory lesions and vascular complications (25).

To date, to the best of the authors’ knowledge, there are no studies regarding histopathological pituitary alterations in leishmaniasis; however, endocrine disorders of primary pituitary origin have already been reported in humans (26) and experimental models (27). The BBB is permeable in the pituitary region, and there are studies of nervous tissue involvement in dogs with CanL with a description of CD3^+^ T lymphocyte involvement (28–31). The aim of this study investigated the presence of *Leishmania* spp. amastigotes and CD3^+^ T lymphocytes in different regions of the pituitary gland of dogs with CanL.

## 2. Materials and methods

### 2.1. Ethics

All the procedures and methods used in this study were performed in accordance with the Committee on Ethics and Animal Experimentation of School of Veterinary Medicine, São Paulo State University, UNESP, Araçatuba, Brazil (FOA-0354-2021).

### 2.2. Animals

Twenty-six dogs (15 males and 11 females), aged between 1 and 7 years, and varied breeds, came from the Veterinary Hospital of FMVA-UNESP and the Zoonoses Control Center of Araçatuba, State of São Paulo, Brazil.

### 2.3. Diagnosis of visceral leishmaniasis

The serological diagnosis was obtained by Enzyme-Linked Immunosorbent Assay (ELISA; cut-off >0.270) according to Lima et al. (32) and/or direct cytological investigation of popliteal lymph node aspirate (fine needle aspiration - FNA).

### 2.4. Experimental design

Twenty-one dogs that were naturally infected with *Leishmania* ssp. and not vaccinated or treated for CanL were included in the infected group (G1). These animals were euthanized following the LVC Surveillance and Control Manual of the Ministry of Health (33) and Resolution 1000/2012 of Conselho Federal de Medicina Veterinária (34), which guide and allows the euthanasia of dogs after the diagnosis was confirmed by parasitological examination and/or serological assay. Moreover, five uninfected dogs with no history of neurological impairment, whose euthanasia was performed “*in extremis*” after suffering severe trauma as a result of a car accident, were included in the control group (G2).

### 2.5. Sample collection

Necropsies were performed after confirming the death of the animal. They were evaluated in the macroscopic examination, the absence or presence of changes to determine the clinical staging of the disease, according to Mancianti et al. (35) and described by Reis et al. (36) is available in Supplementary Table S1. According to the standard procedure, samples were collected from various organs, including the pituitary gland. After removal of the encephalon, the pituitary was accessed at the pituitary fossa and the base of the sphenoid bone and sella turcica were removed as a block with the gland. This block was fixed whole in 10% buffered formalin for 24–48 h. The gland was then separated and processed in individual cassettes. After paraffin embedding, the histological sections stained with HE were analyzed. The distinction of the anterior and posterior lobule at macroscopy, was sometime challenging. Because that, some samples had to be reincluded to correct the orientation of the histological sections. Only samples with all the three regions of pituitary was evaluated. All samples were fixed in 10% buffered formalin and processed for paraffin embedding. Histological sections (5 μm) were stained with hematoxylin and eosin (HE) and subjected to immunohistochemical staining (IHQ) (37).

### 2.6. Immunohistochemistry

Immunohistochemistry was performed to detect TCD3 lymphocytes and search for amastigotes of *Leishmania* spp. using the primary antibodies listed in Table 1. We used heterologous hyperimmune serum from mice experimentally infected with *Leishmania* (*V*.) *shawi* (strain 15,789) as the primary antibody for detecting *Leishmania* spp. (kindly provided by Prof. Dra. Márcia Dalastra Laurenti, Laboratory of Pathology of Infectious Diseases of the FMUSP), and anti-CD3 antibody (A0452, Dako) for T lymphocyte staining. Histological sections of lymphoid tissue (popliteal lymph node) from a dog positive for CanL, is shown in Supplementary Figure S1; and human tonsil for TCD3^+^ lymphocytes were used for positive control of the reactions.

**Table 1:** List of antibodies, concentrations, antigen retrieval methods, and chromogens used in immunohistochemistry reactions.

Target	Antibody	Dilution	Antigen retrieval	Chromogen
T lymph	Anti-CD3, (A0452, Dako)	1:100	Trypsin, pH 7.8, 37°C for 30 min in the oven	DAB (K3468, Dako)
*Leishmania* spp.	HMS	1:1000	Citrate buffer pH 6.0, 100°C for 30 min in the steam	DAB (K3468, Dako)

T lymph, T lymphocytes; Trypsin, (T-7409, SIGMA); DAB, 3,3′-diaminobezidine; HMS, hyperimmune mouse serum.

For deparaffinization and hydration, the histological sections were subjected to consecutive 5-min baths in xylene I, II, and III, followed by hydration in alcohol at decreasing concentrations (100, 95, 70, and 50%) and distilled water. To search for amastigotes, antigen retrieval was performed (Table 1), followed by three washes with phosphate-buffered saline (PBS 1%, pH = 7.4). The peroxidase block was performed by immersing the sections in a solution of 50 mL of methanol, 50 mL of distilled water, and 2 mL of hydrogen peroxide for 30 min at ambient temperature (25°C). To detect TCD3^+^ lymphocytes, endogenous peroxidase was blocked, followed by antigen retrieval. After washing three times in PBS, the sections were incubated at ambient temperature (25°C) with a blocking buffer containing 3 g of powdered milk (Nestlé, Brazil) dissolved in 100 mL of buffered saline (pH 7.4) for 30 min at ambient temperature (25°C) to block nonspecific sites. The primary antibodies were diluted in PBS (pH = 7.2) + fetal bovine serum (1%). Standardized dilutions of the primary antibodies are listed in Table 1.

The primary antibodies were incubated with histological sections overnight for 12–14 h at 4°C in a humidity chamber. For the negative control, a diluent without a primary antibody was added. The slides were then incubated with peroxidase-conjugated secondary antibodies (EnVisionTM FLEX – HRP; Dako, K8010) for 45 min at ambient temperature (25°C). After three washes with PBS (pH = 7.4), they were incubated with a DAB substrate kit (K3468, Dako). After washing with tap water and counterstaining with Harris hematoxylin, the sections were dehydrated, diaphanized, and mounted with transparent resin (Entelan®) and glass coverslips. The raw data of the animals and the results of the tests and examinations evaluated are available in Supplementary Table S4.

### 2.7. Histopathological and immunohistochemical analysis

Histological sections of the pituitary gland stained with HE were observed under a light microscope (Olympus BX 50) to evaluate the presence of amastigote forms of *Leishmania* spp. and inflammatory cells in the regions of the *pars nervosa*, *pars intermedia*, and *pars distalis*. The quantification of inflammatory cells was performed by a semi-quantitative method, using a four-point scale (grade 0–3) modified by Grano et al. (38), to represent the intensity of inflammation. The sections were evaluated by two observers who identified the presence and intensity of inflammation using the following scores, absent (0), mild (1), moderate (2), and marked (3) in each analyzed region.

Immunohistochemical evaluation of TCD3^+^ lymphocytes was performed using an Olympus BX 50 microscope coupled with a camera and computer. Images were captured from five high-power fields (HPF; hot spot) of the regions of *pars intermedia*, *pars distalis*, and *pars nervosa*, with a final magnification of ×400. For manual counting of immunolabeled cells with morphology consistent with that of lymphocytes, ImageJ 1.52 software was used, is shown in Supplementary Figure S2. Intravascular cell markings were excluded from the analysis. The investigation of amastigotes of *Leishmania* spp. immunolabeled was performed under light microscopy, using ×40 and ×100 objectives and final magnification of ×400 and ×1,000 in all of the above regions.

### 2.8. Statistical analysis

The Shapiro–Wilk test was used to assess data normality. Differences between groups were determined using the Mann–Whitney test for non-parametric data. Spearman’s correlation test was used to correlate the inflammation score with clinical staging and mean TCD3^+^ lymphocyte count per region. Statistical significance was set at *p* < 0.05. All statistical analyses were performed using Prism software (v8.0.1, GraphPad, La Jolla, CA, United States).

## 3. Results

### 3.1. Immunohistochemical detection of *Leishmania infantum*

**Figure 1:**
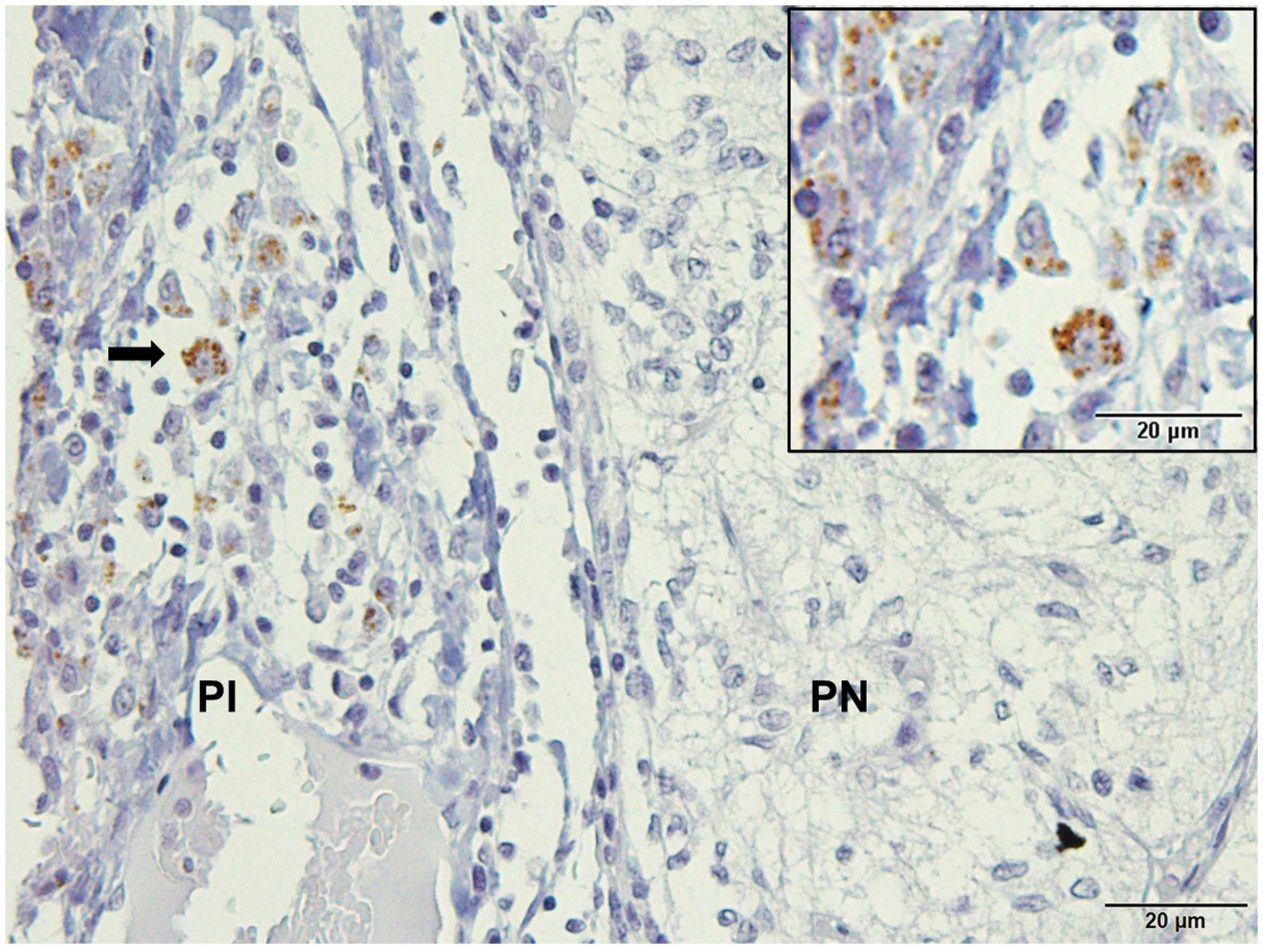
Photomicrography of the pituitary gland of a dog infected with *Leishmania* spp. Immunohistochemistry for detection of amastigotes (arrows). Positive immunolabeling amastigotes in the cytoplasm of macrophages and free in *pars intermedia* (PI) of the adenohypophysis in the marginal area with *pars nervosa* (PN; hyperimmune mouse serum +3,3′-diaminobezidine- DAKO; scale bar = 20 μm).

Immunodetection of *Leishmania* spp. in pituitary sections showed positive staining in 9.52% (2/21) of the samples. Typical amastigote forms were located in the cytoplasm of macrophages and interstitium of the *pars intermedia* of infected dogs (Figure 1).

### 3.2. Clinical staging

Among the dogs in the infected group, 52.38% were classified as symptomatic, presenting with at least three severe macroscopic lesions of the disease, such as diffuse cutaneous alterations (alopecia, ulcers, and furfuraceous dermatitis), ocular (blepharitis and keratoconjunctivitis), marked emaciation, and other clinical signs characteristic of CanL, such as lymphadenomegaly and onychogryphosis. Dogs were classified as asymptomatic, with no evident lesions, and oligosymptomatic, with skin lesions, slight weight loss and lymphadenomegaly, each representing 23.81% of the dogs (Supplementary Table S2).

### 3.3. Histopathological characterization

**Figure 2:**
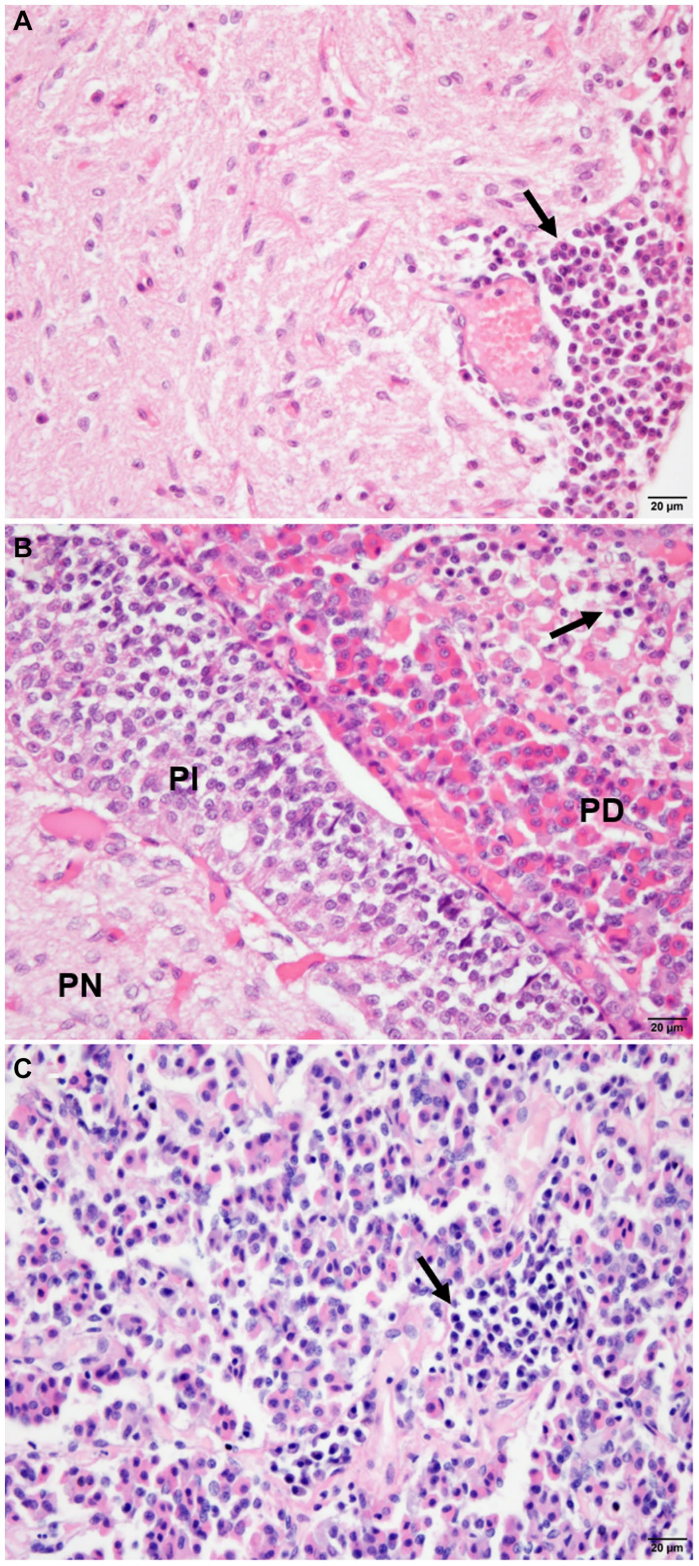
Photomicrograph of the pituitary regions of the dogs (G1), demonstrating the distribution and intensity of inflammation composed of mononuclear cells (arrows). **(A)** Note the inflammatory cells with focal, perivascular distribution in the *pars nervosa* (PN), with moderate intensity. **(B)** Observe inflammatory cells intermingled with chromophilic, acidophilic, and basophilic cells with focal distribution and intensity in *pars distalis* (PD), and absence of inflammation in *pars intermedia* (PI). **(C)** Note the inflammation with focal and moderate distribution in the parenchyma in *pars distalis* (HE staining, bar = 20 μm).

**Figure 3:**
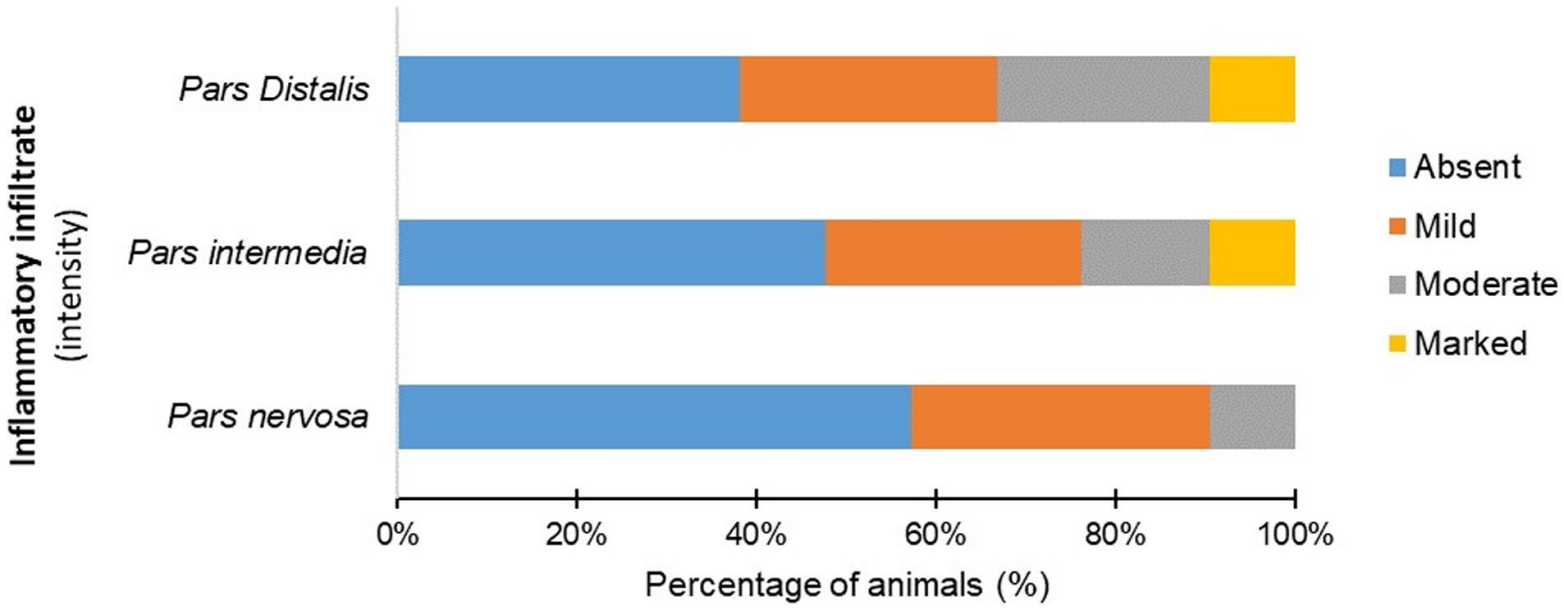
Percentage (%) of infected dogs (G1) according to the intensity of mononuclear inflammatory cells observed in the *pars distalis*, *pars intermedia*, and *pars nervosa*.

The infected animals (G1) showed an inflammatory infiltrate composed of lymphocytes, plasma cells, and rare macrophages in the pituitary regions evaluated (Figure 2; Supplementary Table S3). Mononuclear cells were observed in the *pars intermedia* of 11 animals (11/21), which was classified as discrete in 28.87% (6/21), moderate in 14.29% (3/21), and accentuated in 9.52% (2/21). In *pars distalis*, the presence of inflammatory cells was observed in 13 animals (13/21), with discrete intensity in 28.57% (6/21) of the animals, moderate in 23.80% (5/21), and accentuated in 9.52% (2/21). In the *pars nervosa*, inflammation was evident in nine animals (9/21), with discrete intensity in 33.33% (7/21) and moderate intensity in 9.52% (2/21) (Figure 3). The group of uninfected animals (G2) showed minimal changes, with the discrete presence of focal mononuclear cell clusters in the *pars distalis* in 40% (2/5) of the animals and 20% (1/5) in the *pars intermedia*. The *pars nervosa* showed no inflammation in 100% (5/5) of animals in the control group. There was no correlation between the clinical stage of leishmaniasis and inflammation score in any of the pituitary regions in the infected group (G1): *pars nervosa* (*p* = 0.8565), *pars intermedia* (*p* = 0.1986), and *pars distalis* (*p* = 0.8284).

Besides inflammation no changes such as hyperplasia was observed at adenohypophysis. And also in *pars nervosa*, a glial reaction (gliosis) was observed in 52.38% (11/21) of the dogs in the infected group.

### 3.4. Immunostaining for TCD3^+^ lymphocytes

**Figure 4:**
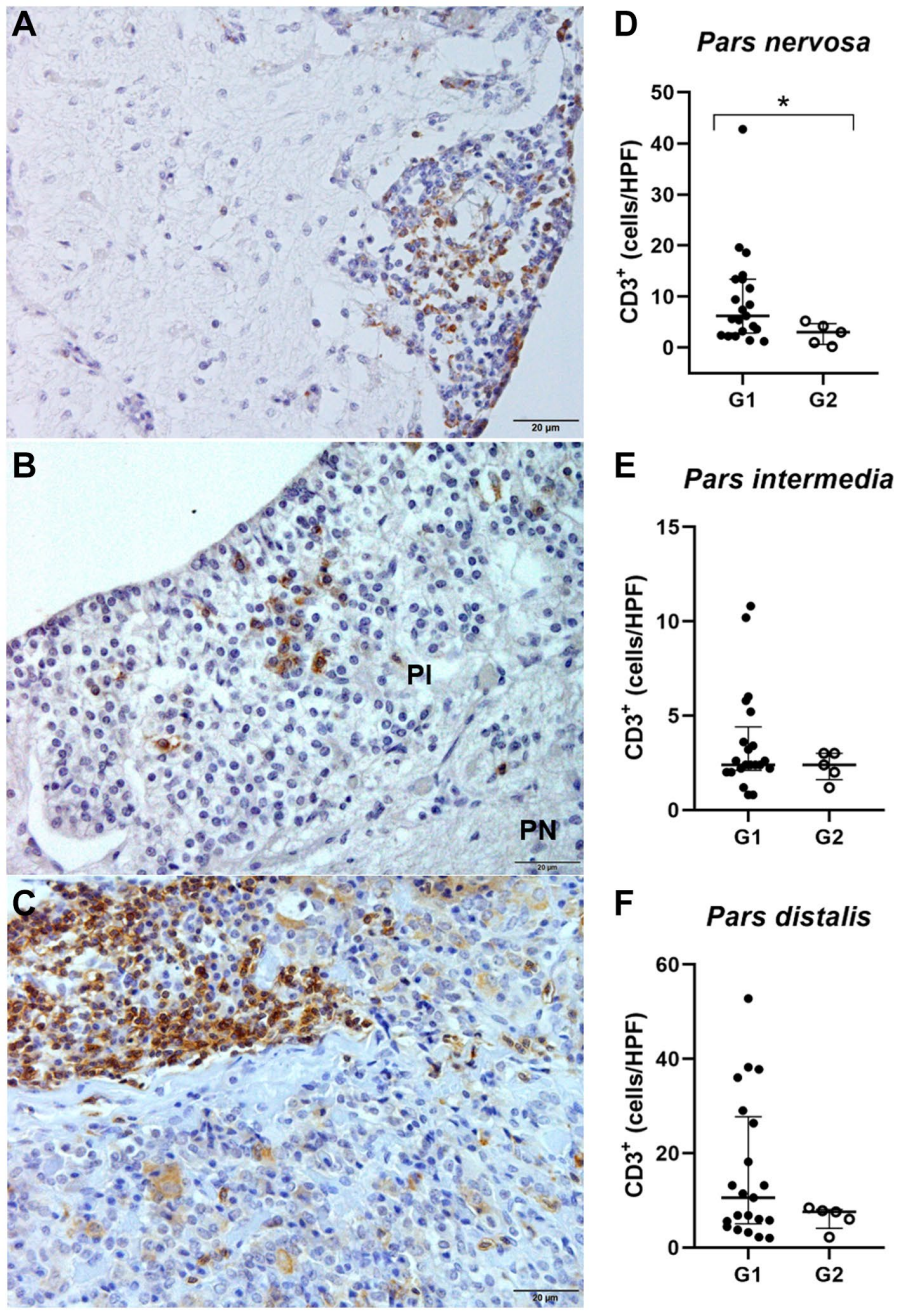
**(A,C,E)** Photomicrograph representing the *pars nervosa* (PN), *pars intermedia* (PI), and *pars distalis* regions of the pituitary gland of a dog infected with *Leishmania* spp., immunohistochemistry for detection of CD3^+^ T lymphocytes revealed with DAB DAKO (scale bar = 20 μm). **(B,D,F)** Dispersion graphs showing the number of TCD3^+^ lymphocytes in the pituitary regions of dogs in group G1 (*n* = 21) and G2 (control *n* = 5). The horizontal lines represent the median and interquartile range values. **(B)** The number of TCD3^+^ cells was higher in the *pars nervosa* (*p = 0.0357). **(D,F)** No difference in the *pars intermedia* (*p* = 0.4935) and *pars distalis* (*p* = 0.4000).

Identification and quantification of immunolabeled TCD3^+^ lymphocytes were performed in all analyzed regions of the pituitary gland of infected (*n* = 21) and control (*n* = 5) dogs. The significant differences between groups was determined by region, with a significant difference between G1 and G2 in the *pars nervosa* (*p* = 0.0357) and no significant difference between G1 and G2 in the *pars intermedia* (*p* = 0.4935) and *pars distalis* (*p* = 0.4000; Figure 4).

**Figure 5:**
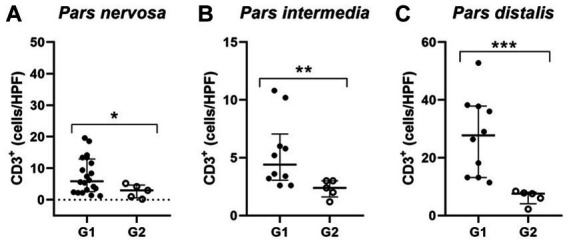
Dispersion graphs showing the number of TCD3^+^ lymphocytes in the pituitary regions of G1 subpopulations. The horizontal lines represent the median and interquartile range values. **(A)** In the *pars nervosa* evaluation, the animal with the highest number of TCD3^+^ cells was removed from the infected dogs (G1; *n* = 20), and when compared to the control group (G2; *n* = 5), the statistical difference was maintained (* *p* = 0.0441); **(B,C)** the *pars intermedia* and *pars distalis* evaluation resulted in a subpopulation of ten infected dogs (G1; *n* = 10) with higher numbers of TCD3^+^ cells compared to the control group (G2; *n* = 5) being selected. This subpopulation of dogs showed significantly increased TCD3^+^ cells, respectively (** *p* = 0.0070) and (*** *p* = 0.0007).

Because these animals are naturally infected, observations in variations in the distribution and number of these cells among individuals are expected, considering that the time since infection is unknown. Furthermore, to homogenize the samples in relation to the number of CD3^+^ T lymphocytes found in the control animals, we determined the median number of CD3^+^ in the respective regions of the pituitary gland of the control dogs and established a cut-off. Therefore, we selected a subgroup of 10 animals within the infected animals that contained a number of CD3^+^ above the average of the control animals. This subpopulation of animals from group G1, when compared to G2, showed a significant difference compared to G2 in the number of TCD3^+^ lymphocytes observed in the *pars intermedia* (**p = 0.0070) and *pars distalis* (***p = 0.0007). Furthermore, in the *pars nervosa* of infected dogs, the difference between G1 and G2 was observed (*p = 0.0441), even with the removal of a single dog that had the highest number of TCD3+ cells (*n* = 20; Figure 5).

**Figure 6:**
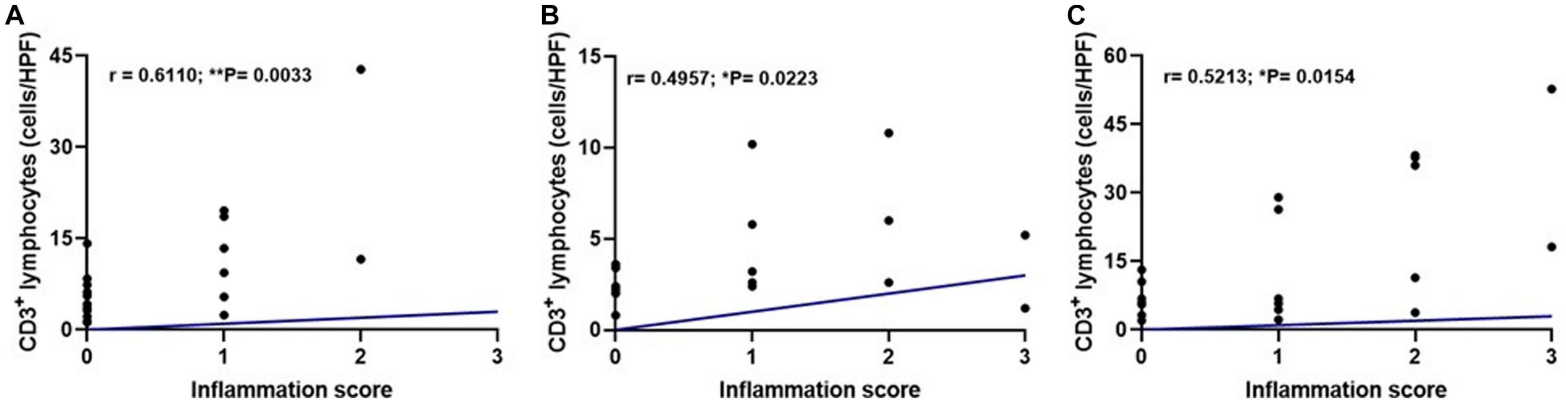
Dispersion graphs showing a moderate positive correlation between inflammation score and median TCD3^+^ lymphocyte count per pituitary region in the infected group (G1, *n* = 21) using Spearman’s test. **(A)** *Pars nervosa* (*r* = 0.6110; **p = 0.0033). **(B)** *Pars intermedia* (*r* = 0.4957; *p = 0.0223). **(C)** *Pars distalis* (*r* = 0.5213; *p = 0.0154).

We performed a correlation test to evaluate if the intensity of inflammation was associated with the increase in TCD3^+^ lymphocytes. A correlation was found between these parameters in the three pituitary regions: *pars nervosa* (**p = 0.0033), *pars intermedia* (*p = 0.0223), and *pars distalis* (*p = 0.0154; Figure 6).

## 4. Discussion

Although CanL is recognized as a serious systemic disease, few studies have described its neurological involvement and complications, including pituitary involvement. We found diffuse inflammation with the presence of CD3^+^ T lymphocytes located mainly in the *pars distalis*. We also detected amastigotes of the parasite in the organs of two animals, which is relevant considering the tropism of the parasite for various organs and its ability to promote an organ-specific immune response. These findings suggest that immune response might promote disturbances in hormone production in the pituitary gland, and consequently in all its target organs, affecting the clinical condition of the animal or similar.

Immunohistochemical evaluation of the pituitary gland of dogs with CanL revealed the presence of *Leishmania* spp. amastigotes in two animals. Owing to the small number of amastigotes in the pituitary tissue, parasite investigation was ineffective on HE-stained sections. However, using immunohistochemistry, immunolabeled amastigotes were observed within and outside the cytoplasm of macrophages in the *pars intermedia* region of the two dogs. This is the first study of amastigotes of *Leishmania* spp. in the pituitary gland of dogs, similar to the encephalon. Studies on the identification of amastigote forms of *L. infantum* in the choroid plexus and meninges of naturally infected dogs are limited (28, 29, 31, 39, 40).

Similar to the findings of studies performed on the brains of dogs with CanL, inflammation was not related to the presence of amastigotes in the tissue (30, 41–43). One hypothesis would be that chronic systemic inflammation resulting from *L. infantum* infection, with production of cytokines and chemokines, could alter barrier permeability (44–46). Also, the formation of immune-complexes resulting in vasculitis, should be considered, particularly in the case of the pituitary, which is a very vascularized organ and presents more permeable points (47, 48). However, the nervous tissue is also affected in dogs with CanL, with the activation of glial cells, which may be directly related to the modulation of the local inflammatory response and alteration of barrier permeability (46).

In addition to amastigotes, we also observed the presence of an inflammatory infiltrate composed of mononuclear cells (e.g., lymphocytes, plasma cells, and macrophages). There was a moderate significant correlation between inflammation intensity and the number of TCD3^+^ lymphocytes in the analyzed pituitary regions, as demonstrated by immunohistochemical detection. An increased number of TCD3^+^ lymphocytes was observed in *pars nervosa* in the infected group. Some works referenced in the literature, as works performed by our group highlight the predominance of T lymphocytes (49, 50). Cytometry would be the most appropriate method for the characterization and quantification of T lymphocyte phenotypes. However, the small size of the pituitary gland prevented the histological, immunohistochemical and flow cytometry evaluation.

Lymphocytes in the pituitary gland have been described in dogs with concomitant diseases, such as multicentric lymphoma, osteoarthritis, nodular hyperplasia in the *pars distalis*, and pituitary adenoma. The composite cellular response, with a predominance of TCD3^+^ cells, may characterize subclinical lymphocytic hypophysitis (9). In humans, the cause of lymphocytic hypophysitis remains unknown, but based on the nature of the inflammation, T lymphocytes are crucial for the development of the disease, and therefore the hypothesis is that this disorder is of immune-mediated origin (4, 51–53). Immune-mediated lesions are reported in dogs with CanL, especially those seen in highly vascularized organs such as eyes and kidneys (47, 54, 55).

In the pituitary gland of dogs in this study, the highest inflammation intensity was observed in *pars distalis* and *pars intermedia* of the adenohypophysis. In humans with lymphocytic hypophysitis, adenohypophysis is the most affected area (50). The autoimmune process possibly targets specific pituitary cell subtypes, possibly causing damage to corticotrophic hormone (ACTH), FSH/LH, or TSH-secreting cells in the adenohypophysis (52, 53, 56).

In humans, similar cases of lymphocytic hypophysitis have been observed, highlighting that these inflammatory cells in the *pars distalis* and *pars nervosa* should be considered pathological (51, 57, 58). Furthermore, lymphocytic hypophysitis has been associated with hypopituitarism, secondary hypoadrenocorticism, and impaired ACTH and cortisol production (7). It is also considered a probable cause of sudden death in dogs (2, 3, 6, 9).

Unfortunately, as this study was performed on animals admitted for necropsy, we were unable to perform hormonal measurements. Future investigations using a larger number of dogs and correlating the occurrence of hypophysitis with hormonal changes would be valuable to improve the significance of the results presented.

Plasmocytes have also been observed to be involved in inflammation in the pituitary glands of dogs in this study. This cell type was observed by HE staining of pituitary tissue samples, and although the presence of plasma cells is described in cases of hypophysitis in humans, this number is apparently lower in dogs than in TCD3^+^ lymphocytes. These cells were not immunohistochemically characterized in our study. Similar to the evaluations performed in human hypophysitis, further studies are needed to understand the function of these cells in cases of lymphocytic hypophysitis (9, 56).

No relationship was observed between the clinical signs of CanL presented by the dogs following the clinical staging proposed by Reis et al. (35, 36) and the intensity of inflammation in the pituitary tissue. These data suggest that pituitary gland inflammation is not related to the clinical stage of CanL. Our findings corroborate those of other studies analyzing changes in the brain in animals with CanL, where there was no correlation between the clinical stage of the disease and the intensity of inflammation in the nervous tissue (30, 38, 41, 50). However, the dogs evaluated were naturally infected and, therefore, a heterogeneous group in relation to the infection stage, making it difficult to understand the evolution of inflammatory changes in the pituitary gland.

The anti-*Leishmania* immune response includes the production of various cytokines (24), such as TNF, which can regulate the endocrine system, thereby being able to inhibit the secretion of pituitary hormones such as TSH (59) and gonadotrophic hormone (60).

Studies of endocrine abnormalities in humans with visceral leishmaniasis, such as dysfunction in antidiuretic hormone (ADH) secretion and alterations in the hypothalamic–pituitary–adrenal, pituitary-thyroid, and pituitary-gonadal axes (26), have been reported; however, they require further investigation in dogs. We can infer that the occurrence of hypophysitis in CanL may also result in the production of cytokines that may have local or systemic action and may interfere directly or indirectly with the production of pituitary hormones and contribute to the positive or negative modulation of the host response to infection (26, 45, 59, 60). Further studies are needed to elucidate the mechanisms and pathogenesis of CanL in the pituitary gland and its consequences on the patient’s clinical condition. Possible mechanisms involved in the complication of neurological lesions in dogs are suggested, such as direct effect of the parasite, disease-related immune-mediated lesions, and vasculitis caused by indirect effects of leishmaniasis (25, 47).

This is the first study of the presence of *Leishmania* spp. amastigotes in the pituitary gland and hypophysitis in dogs infected with *Leishmania* spp. The presence of TCD3^+^ lymphocytes and/or parasites in the pituitary region presuppose dysfunction of the gland and possible worsening of the patient’s clinical status, compromising the efficiency of treatment and prognosis.

## Data availability statement

The original contributions presented in the study are included in the article/Supplementary material, further inquiries can be directed to the corresponding author.

## Ethics statement

The animal studies were approved by Committee on Ethics and Animal Experimentation of School of Veterinary Medicine, São Paulo State University, UNESP, Araçatuba, Brazil (FOA-0354-2021). The studies were conducted in accordance with the local legislation and institutional requirements. Written informed consent was obtained from the owners for the participation of their animals in this study.

## Author contributions

EF: project execution, data collection and curation, analysis of results, and writing the draft. CG: data collection and processing, critical revision of the text. GJ: proofreading and editing significant parts of the work. KM: data collection and processing, statistical analysis. GuM: statistical analysis and critical revision of the text. TW: critical revision of the text. GiM: coordination of the research activity planning and execution, acquisition of financial support. All authors contributed to the article and approved the submitted version.

## Funding

This study was financed in part by the Coordenação de Aperfeiçoamento de Pessoal de Nível Superior, Brasil (CAPES), finance code 001.

## Conflict of interest

The authors declare that the research was conducted in the absence of any commercial or financial relationships that could be construed as a potential conflict of interest.

## Publisher’s note

All claims expressed in this article are solely those of the authors and do not necessarily represent those of their affiliated organizations, or those of the publisher, the editors and the reviewers. Any product that may be evaluated in this article, or claim that may be made by its manufacturer, is not guaranteed or endorsed by the publisher.
