# Supplementary material for: Lymphocytic hypophysitis in dogs infected with Leishmania spp
Source: Front Vet Sci. 2023 Sep 14;10:1208919. doi: 10.3389/fvets.2023.1208919 (PMC10537919; doi:10.3389/fvets.2023.1208919)
Supplement: Supplementary file 1 [file Data_Sheet_1.docx]

Supplementary Material

Lymphocytic hypophysitis in dogs infected with *leishmania* spp.

**Edenilson Doná Frigerio, Cecilia de Castro Guizelini, Giulia Gonçalves Jussiani, Karen Santos Março, Guilherme Dias de Melo, Tatiane Terumi Negrão Watanabe, Gisele Fabrino Machado***

*** Correspondence:** Corresponding Author: gisele.fabrino@unesp.br

**Supplementary Table 1.** Classification was established according to the clinical staging of CanL, according to Mancianti et al. (1988).

| **Classification** | | **Observation** |
| --- | --- | --- |
| 0 | Asymptomatic | Absence of signs and symptoms related to *Leishmania* infection |
| 1 | Oligosymptomatic | Lymphoid adenopathy, slight weight reduction, and/or opaque hair |
| 2 | Symptomatic | All or some severe signs of the disease, i.e., skin changes (alopecia, dermatitis furfuracea, ulcers), onychogryphosis, keratoconjunctivitis, and hind limb rigidity |

**Supplementary Table 2.** Clinical staging of infected dogs (G1) into asymptomatic, oligosymptomatic, and symptomatic.

| **Clinical staging** | **N** | **%** |
| --- | --- | --- |
| Asymptomatic | 5 | 23,81 |
| Oligosymptomatic | 5 | 23,81 |
| Symptomatic | 11 | 52,38 |
| Total | 21 | 100 |

**Supplementary Table 3.** Percentage of infected (G1) and control (G2) dogs that presented with inflammation in the pituitary regions, graded by presence and intensity.

|  | **Pituitary Regions** | **Presence of mononuclear cells** | **Mild** | **Moderate** | **Marked** |
| --- | --- | --- | --- | --- | --- |
| G1  (n21) | *Pars nervosa* | 9 | 7 (33,33%) | 2 (9,52%) | - |
|  | *Pars intermedia* | 11 | 6 (28,57%) | 3 (14,29%) | 2 (9,52%) |
|  | *Pars distalis* | 13 | 6 (28,57%) | 5 (23,81%) | 2 (9,52%) |
| G2  (n5) | *Pars nervosa* | 0 | - | - | - |
|  | *Pars intermedia* | 1 | 1 (20%) | - | - |
|  | *Pars distalis* | 2 | 2 (40%) | - | - |

**Supplementary Table 4.** Experimental raw data.


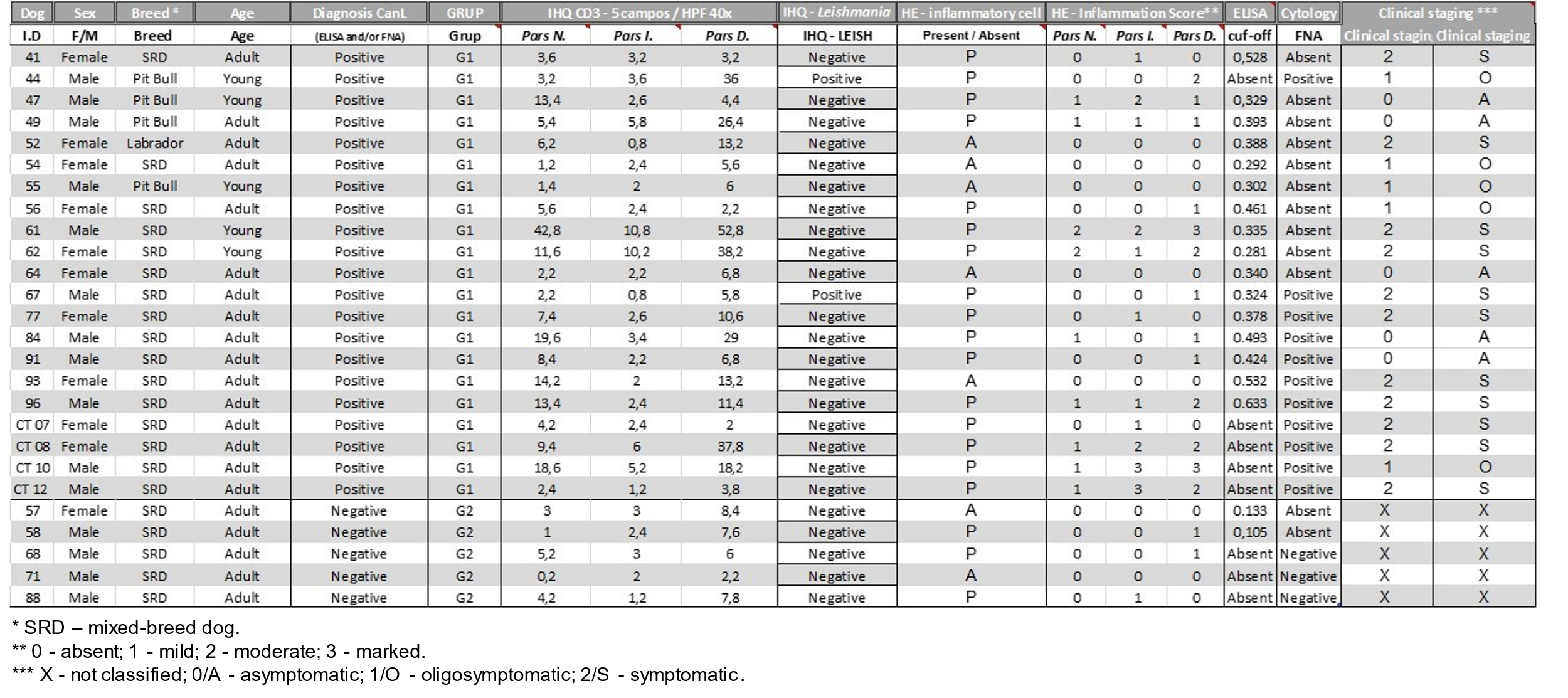


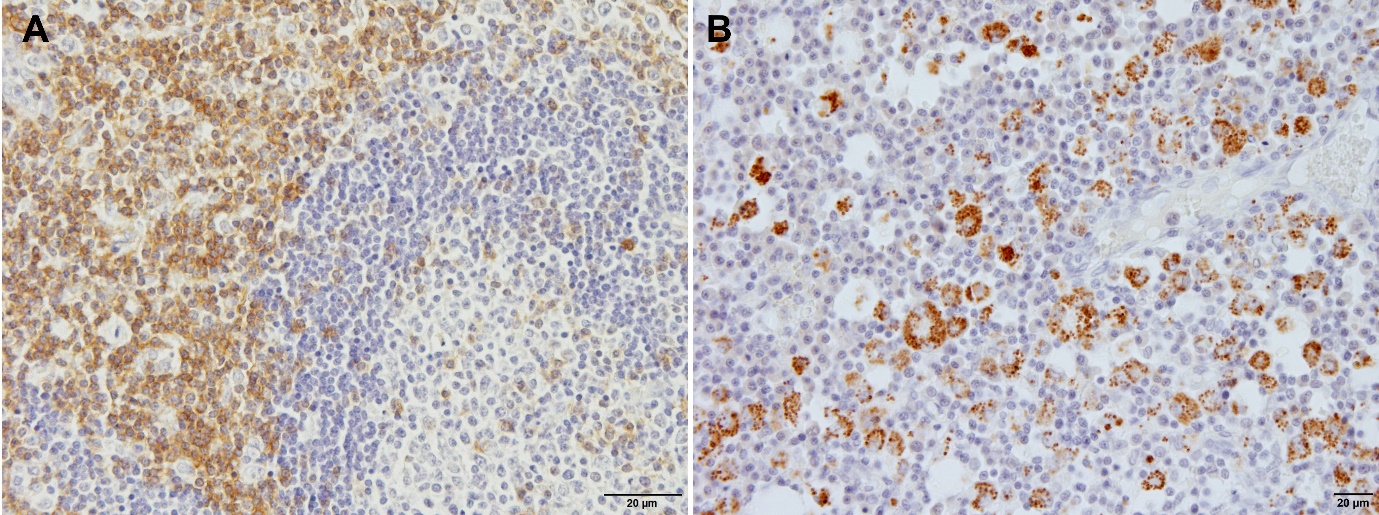


**Supplementary Figure 1.** Photomicrograph representative of the positive control of the immunohistochemistry reactions of histological sections of lymphoid tissue (popliteal lymph node) from CanL-positive dogs revealed with DAB DAKO (scale bar = 20 μm). (A) Detection of CD3^+^ T lymphocytes using an anti-CD3 antibody (A0452, Dako). (B) Immunolabeling for *Leishmania* spp. using heterologous hyperimmune serum from mice experimentally infected with *Leishmania* (V.) *shawi* (strain 15789).


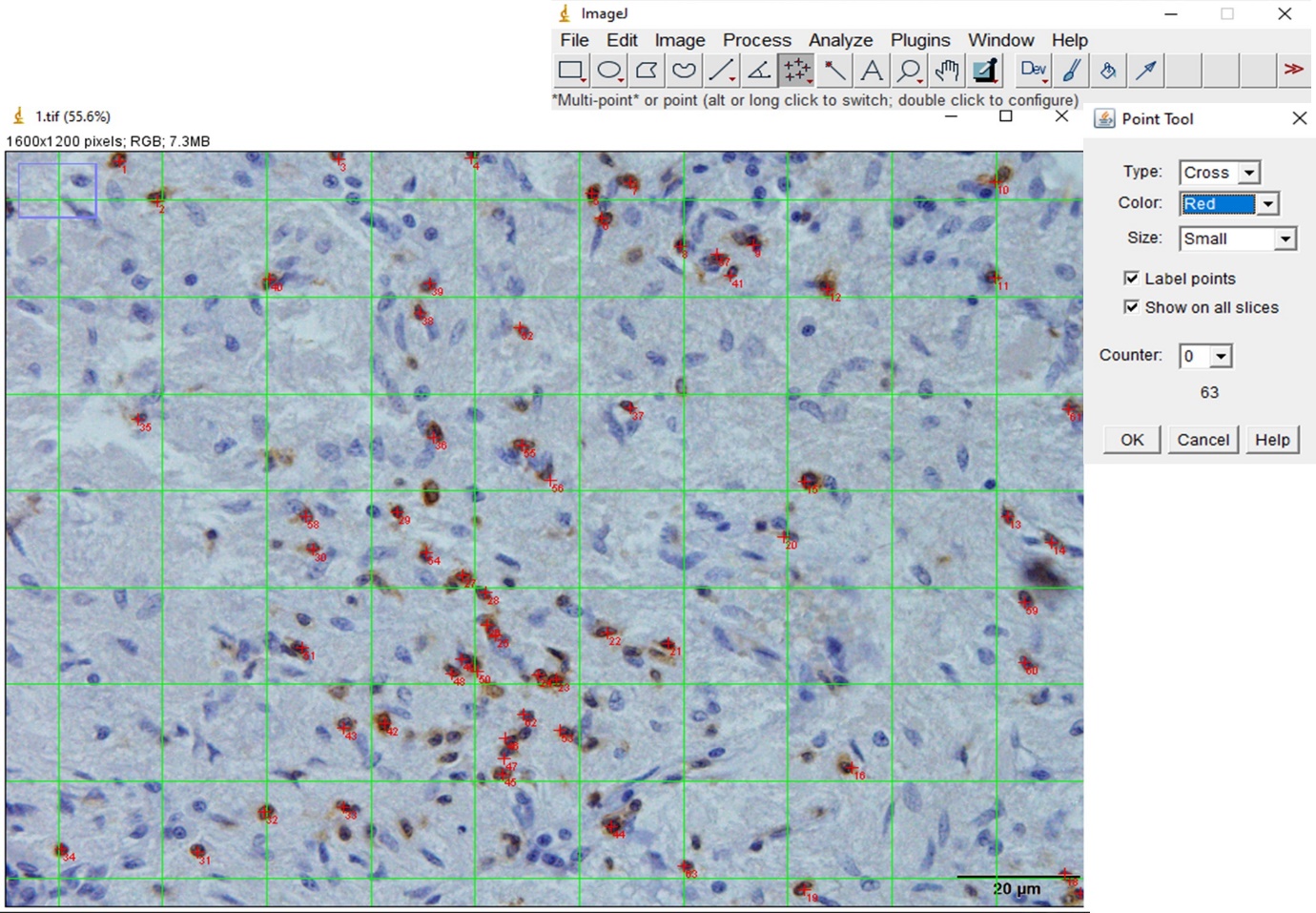


**Supplementary Figure 2.** Demonstration of the TCD3^+^ cell count immunostained with 3,3'-diaminobenzidine (DAKO) developer using ImageJ software, in red counting of selected TCD3^+^ lymphocytes using the software tool, green line grid made by the program to help the counting. Photomicrography was performed using an Olympus BX 50 digital micrograph device coupled to a camera and computer for image capture using Cellsens software (Olympus Life Science) at 40× magnification; hot spot). Bar= 20 µm.
